# Myringoplasty in children: retrospective analysis of 60 cases

**DOI:** 10.11604/pamj.2015.20.82.3620

**Published:** 2015-01-29

**Authors:** Harkani Abdellatif, Rouchdi Youssef, Maliki Omar, Nouri Hassan, Aderdour Lahcen, Raji Abdelaziz

**Affiliations:** 1 ENT Department, Mohammed VI University Hospital, Marrakech, Morocco

**Keywords:** Myringoplasty, child, cartilage

## Abstract

Myringoplasty or type 1 tympanoplasty aims the restoration of the anatomic integrity of the tympanic membrane; it's a very common surgery in otology. The objective was to evaluate the anatomic and functional results of this surgery in children using the retro-auricular approach. Sixty young patients with diagnosis of simple tympanic perforation were evaluated; these patients underwent myringoplasty by a retro-auricular approach (underlay technique) between November 2010 and May 2013. It's a retrospective evaluation of the anatomic and functional results of theses myringoplasties. Mean age at surgery was 8, 5 years old, cartilage was used as graft in our entire patient, closure of perforation was successful in 48 cases (80%), and audiometric results showed functional improvement in 27 (45%) patients, no significant change was noted in the remaining patients. The results of myringoplasty in children seem worse than those in adults. However, a large study with a long follow up is warranted in order to come to definitive conclusions.

## Introduction

Myringoplasty in children is often less successful than in adult, the dysfunction of the Eustachian tube and the elevated incidence of otitis media in the pediatric population are often implicated as the reason for poorer results [1, 2]. Several types of graft can be used, but the cartilage is more suitable for the pediatric population. The present study analyzes the anatomic and functional results of myringoplasty in children using large plate of choncal cartilage.

## Methods

Between November 2009 and May 2013, 60 young patients were operated for tympanic Perforation. The inclusion criteria were: age less than 15 year's old, non marginal tympanic perforation and the absence of otorrhea at surgery. Cases with cholesteatoma and ossicles lesions were excluded. All procedures were done under general anesthesia. retroauricular approach was used in all cases. The underlay technique was used to placing the cartilaginous graft. Data was collected from medical records; the anatomic evaluation was based on otomicroscopic examination 3 and 6 months after surgery. Pure tone audiometry at 500, 1000, 2000, 3000, 4000 Hz was used to evaluate the functional results 3 months and 6 months after surgery. An intact graft at the end of 6th month was considered as an anatomic success and a minimum hearing improvement of 10 db in two consecutive frequencies was regarded as a functional success.

## Results

Our population includes 60 patients, the sex-ratio was 1, 45 (35 males, 24 females), age range varies from 5 to 15 years old (mean age at surgery was 8, 5 years old, 44 had more than 10 years old and 16 less than 10 years old). 55 patients (91,5%) have consulted for recurrent episodes of otorrhea, 15 patients (24%) for earache, 7 patients (11,6%) for resounding hearing loss and 5 patients (8,3%) for tinnitus (Table 1). The otomicroscopic examination shows: central non marginal perforation in 29 patients, posterior perforation in 17 patients and anterior perforation in 14 patients. The tympanic cavity was dry in 27 cases, wet in 18 cases and inflammatory in the 15 remaining cases. The conralateral ear was identified as normal in 32 cases (53,3%), 18 contralateral ears (30%) were perforated, 8 patients presented contralateral tympanic atelectasis and only two patients presented active contralateral otorrhea (Table 2). The preoperative audiometric evaluations were performed 7 days before surgery, they showed: an air-bone gap less than 20dB in 19 cases, less than 30 dB in 26 cases and more than 30 dB in 11 cases. All patients were operated under general anesthesia using the retro-auricular approach; the choncal cartilage was used as graft and placed under the malleus in all cases. The mean follow up was 9 months. Concerning the functional outcomes after 6 month of surgery: improvement was noted in 29cases (48,3%); 22 of them had less than 15 dB of gain, the 7 remaining cases obtained more than 15 dB of gain. No significant changes were noted in 31 patients (Table 3). Concerning the anatomic results: closure of the tympanic membrane was obtained in 48 cases (80%), residual perforations was identified in the other 12 cases. The closure rate was more important in children over 10 years old (70,4% for children over 10 years and 37,5% for children under this age), the closure was also more important for central perforation (65,5%) and in cases with dry taympanic cavity (77,8%) (Table 4). The closure ratio was also influenced by the status of contralateral ear; the ratio was 60, 6% for normal contralaeral ear and only 21, 4% for pathologic contralateral ear.

**Table 1:** Reason for first consultation

Signs	Number of case	rate
Otorrhea	55	91,5%
earache	15	24%
tinnitus	7	11,6%
Hearing loss	5	8, 3%

**Table 2:** Status of contralateral ear

Status of contralateral ear	Number of cases	
	N	%
Normal	32	(53,3%)
Abnormal	28	(46,7%)
Tympanic perforation	18	(30%)
Atelectasia	8	(13,3%)
Active otorrhea	2	(3%)

**Table 3:** Functional outcomes

Air bone gap (dB)	Pre-operative	Postopeartive
0-20	19 (31 ,6%)	32 (53,3%)
21-30	26 (43,3%)	23 (38,3%)
More than 30	11 (18,3%)	5 (8,3%)

**Table 4:** Anatomic results at the sixth month

	Global results	Results according to age	Results according to CL ear	Results according to the status of tympanic cavity	Results according to perforation site
Closure rate	48/60 (80%)	>10: 70,4% (31/44) <10: 37,5% (6/16)	Normal:60,6% Abnormal:21,4%	Dry:77,8% Wet or inflammatory: 22,2%	Central: 65,5% Anterior:35,7% Posterior:100%

## Discussion

The goals for most pediatric tympanoplasty operations are 3 fold: First, the goal is to create an intact tympanic membrane to prevent middle ear contamination and allow unrestricted water activities. Second, the intact tympanic membrane should allow good, serviceable hearing. Finally, an aerated, sound conducting middle ear space should be achieved [1, 2]. Although a healed tympanic membrane was the primary goal of the surgery, it was not the only measure of success [3]. The closure ration in pediatric population varies according literature from 32% to 91% [3, 4], in our study it's estimated at 80%. Age is often cited as a key prognostic factor in evaluation for tympanoplasty in children because Eustachian tube function is known to normalize with advancing age. Some authors recommended a minimum age to consider tympanoplasty [5], ranging from 6 to 8 years. In a meta-analysis of 19 articles by Vrabec et al [6], only increasing age was associated with a statistically higher chance of surgical success, in our data the anatomic results were better in children over 10 years old. In a study of 318 pediatric tympanoplasties in which nearly 60% of patients had abnormal contralateral ears, Chandrasekhar et al [7] found no effect on healing rates. Other investigators have reported the abnormal contralateral ear as a negative prognostic factor in pediatric Tympanoplasty [7], in our study the pathologic contralateral ear seems as a pejorative factor of tympanplasty success. Many otorhinolaryngologists believe that a dry ear is important for graft integration; others think that this is not so important for surgical success. A few studies have shown that a wet ear is a negative prognostic factor for graft integration [7, 8]. Several other studies have found no significant statistical correlation [9]. In our study dry tympanic cavity seems benefic for graft integration. Some authors have mentioned that the perforation site influences the prognosis more than the perforation size [7]; anterior perforations are technically more difficult to access and to place a graft adequately; the blood supply is also poorer. Singh et al [10] showed that the graft integration rate was 34% in anterior perforations, 91% in inferior perforations, and 100% in posterior perforations. Other studies have concluded that the perforation site had no effect on graft integration or the results of hearing, in our study we have better tympanic results with central and posterior perforations comparing to anterior perforations. Techniques for performing tympanoplasty can vary widely by author and institution, and largely reflect the primary surgeon's training and experience, in our study all patients were operated with underlay technique. The cartilage seems more resistant than temporal fascia, the functional outcomes are better with fascia but most authors prefer the cartilage in pediatric population, Some randomized study concluded that there's no significant statistical difference between fascia and cartilage [11].

## Conclusion

Myringoplasty in children seems be influenced by factor such as: patient age, seize and site of perforation, contralateral ear status and the status of the tympanic cavity.
